# Gender and Ocular Manifestations of Connective Tissue Diseases and Systemic Vasculitides

**DOI:** 10.1155/2014/403042

**Published:** 2014-03-17

**Authors:** Maria M. Choudhary, Rula A. Hajj-Ali, Careen Y. Lowder

**Affiliations:** ^1^Cole Eye Institute, 9500 Euclid Avenue, I-10, Cleveland, OH 44195, USA; ^2^Department of Rheumatology, Cleveland Clinic, 9500 Euclid Avenue, A-50, Cleveland, OH 44195, USA

## Abstract

Ocular manifestations are present in many connective tissue diseases which are characterized by an immune system that is directed against self. In this paper, we review the ocular findings in various connective tissue diseases and systemic vasculitides and highlight gender differences in each disease. In rheumatoid arthritis, we find that dry eyes affect women nine times more than men. The other extra-articular manifestations of rheumatoid arthritis affect women three times more commonly than men. Systemic lupus erythematosus can involve all ocular structures and women are nine times more affected than men. Systemic sclerosis is a rare disease but, again, it is more common in women with a female to male ratio of 8 : 1. Polymyositis and dermatomyositis also affect women more commonly than men but no gender differences have been found in the incidence or disease course in the systemic vasculitides associated with antineutrophil cytoplasmic antibody such as granulomatosis with polyangiitis (GPA, formerly known as Wegener's granulomatosis). Finally, Behcet's disease is more common in males, and male gender is a risk factor for Behcet's disease. There is a slight female preponderance in sarcoidosis with female gender carrying a worse prognosis in the outcome of ocular disease.

## 1. Introduction

Many connective tissue diseases have abnormal immune system activity with inflammation in tissues as a result of an immune system that is directed against one's own body tissues (autoimmunity). Ocular inflammation is seen as part of a number of systemic diseases with autoimmune processes heading the list. Inflammation can affect any part of the eye starting from the cornea anteriorly to the retina, uveal tract and sclera posteriorly. In some conditions, uveitis or scleritis is the heralding presentation and in others it determines the need for more aggressive immunosuppressive therapy. The incidence, severity, and disease course of uveitis and scleritis are variable with many factors contributing to the natural history of the disease, including gender, the underlying systemic disease, and the extent of the inflammatory process. The ocular immune process is mediated by T helper cells 1 (TH1). It can be TH1 predominant with proinflammatory cytokines or antibody mediated through TH2 cells. Females have a stronger autoimmune response compared to males with TH1 pathway being more vigorous than TH2 except in pregnancy when TH2 takes over [1]. Studies have shown that administration of sex hormones alters autoimmune processes; estrogen upregulates whereas androgens suppress it [2, 3]. The effect of estrogen however is dose dependent with lower levels being immune-stimulatory and higher levels immune-inhibitory. This has been proposed to be responsible for improvement of certain autoimmune pathologies such as rheumatoid arthritis during pregnancy. Similarly women have higher levels of prolactin and growth hormones compared to males. These pituitary hormones also enhance autoimmunity [1]. The effect of hormonal factor in inflammatory eye disease is complex and is not uniform among all diseases; male patients with Behçet's syndrome have worse ocular prognosis than their female counterpart. Nonhormonal gender differences such as environmental exposures, drugs (more commonly used by one gender than the other), or infectious organisms can also play a role.

Autoimmune diseases affect the eye in different ways. We will summarize the ocular manifestations of the most encountered connective tissue diseases and vasculitides.

## 2. Rheumatoid Arthritis

Rheumatoid arthritis (RA) is an inflammatory arthritis associated with a variety of extra articular manifestations. It is three times more common in women than men. There is a genetic predisposition with more than 90% patients carrying the HLA-DR4 and HLA-DR1 genes [4]. Dry eye syndrome is the most common ophthalmic manifestation; women are 9 times more commonly affected than men. It presents as foreign body/gritty sensation, redness, burning, photophobia, or even fluctuating blurry vision. The severity of dry eye does not parallel underlying rheumatoid arthritis disease activity [5].

Dry eye can be either “aqueous deficient” or “evaporative” type. The aqueous deficient component is more commonly associated with autoimmune conditions. It results from immune mediated destruction of the exocrine glands resulting in decreased tear production by the lacrimal glands, also referred to as secondary Sjogren's syndrome [6]. Gender plays a key role with 90% of the patients being females. Females with primary Sjogren's syndrome (without any underlying autoimmune problem) have elevated levels of antinuclear antibody (ANA) and autoantibodies directed against Ro/SSA and La/SSB autoantigens. This is thought to be secondary to higher estrogen levels in women, which, being immune-stimulatory, result in accelerated humoral and cell mediated responses. On the other hand, the evaporative type of dry eye is secondary to tear film instability and higher rate of evaporation. This is thought to be due to relative androgen deficiency. Androgens play a role at various stages in lipid metabolism. Meibomian glands are target organs for androgens. Decreased levels of androgens therefore lead to altered lipid component of meibomian secretions. This alters tear film composition and leads to evaporative dry eye [7]. Women with primary and secondary Sjogren's syndrome are also relatively androgen deficient [8]. Similarly, men on antiandrogen therapy (such as for prostate cancer) are more commonly affected than others. Therefore, female gender has been identified as a risk factor for the development of dry eye [9]. Whether female patients do worse than men on long term follow-up has yet to be studied.

Scleritis and episcleritis are the second most common ocular manifestation of rheumatoid arthritis with RA being the most common autoimmune etiology associated with scleritis [10]. Scleritis can be chronic and associated with significant pain and morbidity. The patients complain of typical deep intolerable boring eye pain, redness, photophobia, and classic ocular tenderness [11, 12]. It usually indicates active systemic disease and sometimes can be the heralding sign. Depending on anatomical location scleritis is classified as anterior or posterior. The former is further subdivided into diffuse, sectoral, nodular, or necrotizing variants with diffuse anterior scleritis being the most prevalent [11]. Significant morbidity is associated with the necrotizing variant, more commonly seen in patients with rheumatoid arthritis than other autoimmune diseases. Slit lamp examination shows a bluish hue in areas of scleral thinning (coming from underlying choroid). On the other hand scleromalacia perforans represents the other end of the spectrum characterized by a quiet eye on exam with significant thinning of the sclera and even rupture [13].

The conjunctiva and episcleral tissue can be similarly involved. Episcleritis usually presents with painless red eye and preserved vision. Some patients complain of minimal discomfort. On exam dilated vessels are seen superficial to the sclera that are relatively mobile and blanch with phenylephrine instillation [14]. No gender differences have been reported in the disease course of scleral and episcleral inflammation associated with rheumatoid arthritis.

Rheumatoid arthritis is the most common autoimmune disease to affect the cornea. Patients typically complain of pain, photophobia, excessive lacrimation, and blurred vision. The cornea does not have its own blood supply and is inaccessible to the inflammatory leukocytes directly. This relatively protects it from immune mediated damage except for the periphery that derives its blood supply from the surrounding episclera. This can cause perilimbal corneal involvement referred to as peripheral ulcerative keratitis (PUK). PUK is often also accompanied with surrounding scleritis. Activation of tissue collagenases results in digestion of corneal stroma with potential loss of the eye [15]. PUK and resultant corneal melt can be seen in a variety of systemic immune destructive processes and is not specific to RA.

Presence of PUK or necrotizing scleritis in patients with rheumatoid arthritis indicates severe systemic disease necessitating immunosuppressive therapy [16].

## 3. Systemic Lupus Erythematosus

Systemic lupus erythematosus (SLE) is an autoimmune disorder that causes immune complex mediated tissue damage affecting a variety of organ systems [17]. It results from interplay of genetic, environmental, and hormonal factors. Gender plays a key role with women being nine times more commonly affected than men. It most commonly affects Asians and African women in their reproductive years [18]. Significant hormonal alterations such as pregnancy and postpartum can cause worsening of disease activity because of increase in the levels of estrogen and progesterone both of which upregulate the TH2 mediated pathway resulting in increased antibody synthesis. Supplemental estrogen in the form of contraceptive pills or hormone replacement therapy has also been proposed to increase the risk of disease flares [19, 20]. Male androgens offer relative protection. Patients with SLE have lower levels of dehydroepiandrosterone (DHEA) and testosterone compared to age matched controls irrespective of the gender [21, 22]. Hypoandrogenism in men has therefore been proposed to increase the incidence and worsen severity of disease activity [23, 24]. The recently revised classification criteria of SLE referred to as the Systemic Lupus International Collaborating Clinics (SLICC) criteria require the presence of at least four of the seventeen criteria for classification of the disease; these should include at least one clinical criterion and one immunologic criterion or biopsy-proven lupus nephritis [25].

Ocular pathology secondary to SLE includes a wide range from the adnexa to the retina and optic nerve. Similar to RA, dry eye syndrome is the most common presentation. SLE is the most common underlying autoimmune pathology in patients with secondary Sjogren's syndrome [26]. It is more frequent in SLE than rheumatoid arthritis and affects more than half of the patients [27, 28]. As in RA, female gender is a risk factor for dry eye syndrome in SLE patients and this is attributed to both the presence of estrogen and lack of androgens. Clinical findings range from abnormal tear film and corneal epithelial changes such as punctate epithelial erosions to more severe forms such as corneal scarring and ulceration. Peripheral ulcerative keratitis as seen in rheumatoid arthritis is uncommon [29, 30].

Retina is the second most commonly involved structure with significant visual morbidity resulting from vascular thrombosis. It can affect as high as a third of all patients with SLE depending on the patient population [29]. The pathogenesis is thought to be an immune mediated endothelial damage and a hypercoagulable milieu leading to vasoocclusive retinopathy. Dilated fundus examination findings can range from cotton wool spots, microaneurysms, and exudates to severe nonperfusion changes such as neovascularization and vitreous hemorrhage [31]. Retinopathy parallels systemic disease activity and simultaneous central nervous system involvement is seen in a vast majority of patients. Significant vision loss as well as increased mortality has been reported in patients with severe retinal vasculitis [32, 33]. Therefore, the presence of retinopathy warrants aggressive systemic therapy even if no obvious signs of systemic disease are seen. Patients with anti-phospholipid antibody are thought to be at a higher risk for developing retinal vasculitis than those who do not carry this antibody [34]; of all the anti-phospholipid antibodies, lupus anticoagulant is associated with the highest risk of arterial and venous thrombosis and resultant vasculitis [35]. Male patients though less commonly affected by SLE have a higher incidence of anti-phospholipid antibodies than females and therefore a higher risk for thrombosis. Male gender has been proposed as a predictor for poor prognosis for extra ocular organ involvement [36]. It is thought that male patients have a higher incidence and more severe retinopathy than their female counterparts, although there have not been epidemiologic studies comparing the ocular manifestations between genders [37, 38].

Scleritis and episcleritis are also common in patients with SLE, but at a less frequent rate than in patients with RA. The pathogenesis is thought to be immune complex mediated tissue destruction of affected tissue [39, 40]. No apparent gender disparities have been reported in terms of incidence or severity of scleral involvement in this patient population. Scleritis can be chronic, relapsing, and sometimes associated with active systemic disease necessitating systemic immunosuppressive therapy [41].

Neuroophthalmic manifestations such as optic neuritis, ischemic optic neuropathy, and chiasmopathy are seen in about 1% of patients with SLE [42]. Patients typically present with painless loss of vision, pupillary defects, and loss/impaired color vision. This can sometimes be the initial manifestation of the disease making the diagnosis difficult. Examination can show disc pallor or edema. This warrants long-term use of systemic immunosuppressive therapy [43, 44]. Given the small population size, no studies are available to comment on gender differences if they exist.

## 4. Systemic Sclerosis

Systemic sclerosis is a rare connective tissue disorder that is characterized by abnormal fibroblast proliferation leading to deposition of extracellular matrix in the skin, blood vessels, and viscera. This results in stiffening of the connective tissue structures in the skin and body organs. Like other autoimmune diseases, it is characterized by interplay of inflammatory cytokines and antibodies [45]. It is more common in women with female to male ratio being 8 : 1 [46]. Most commonly reported ocular pathologies include eye lid stiffness that is seen in more than 50% of the cases and results from deposition of type I collagen in the dermis [47]. Keratoconjunctivitis sicca is the second most common problem seen in 50% of the affected patients [48]. Cases of conjunctivitis, episcleritis, anterior uveitis, and hypertensive retinopathy have also been reported [46, 48–50].

## 5. Polymyositis and Dermatomyositis

As their names suggest polymyositis and dermatomyositis are a group of autoimmune diseases characterized by inflammation of the skeletal muscles; when there is dermatologic involvement, the condition is referred to as dermatomyositis. Genetic factors play an important role and a strong association has been identified with human leukocyte antigens HLA-B8 and DR3 and DR52 [51, 52]. Laboratory testing usually shows elevated creatinine kinase and aldolase levels along with other inflammatory markers [53]. The histopathologic features in dermatomyositis include deposition of immune complexes in a vascular distribution in more than three quarters of the cases. Vasculitis is more commonly seen in the pediatric population [54, 55].

Inflammatory myositis is relatively rare compared to other rheumatologic diseases with around 5 new cases per million being reported annually [56]. Women are more commonly affected than men. Around 15% of the cases are associated with an underlying malignancy [57].

Heliotrope rash or purplish discoloration of the eyelids is the most common ocular manifestation associated with dermatomyositis. Pediatric case reports exist about retinal vasculitis with dermatomyositis [58–60]. Occasional cases of internuclear ophthalmoplegia have also been reported [61, 62]. No gender differences have been observed in the incidence or severity of ocular problems in this patient population.

## 6. Systemic Vasculitides

Classically, the vasculitic syndromes have been classified by the predominant sizes of the blood vessels most commonly involved [63].

Revisions in the commonly used terms for the various vasculitides have been proposed by the 2012 International Chapel Hill Consensus Conference (CHCC) on nomenclature of vasculitides [64].

Notably, the 2012 Chapel Hill Consensus Conference adopted the term antineutrophil cytoplasmic antibody (ANCA) associated vasculitis (AAV) for these three disorders: microscopic polyangiitis (MPA), granulomatosis with polyangiitis (GPA instead of Wegener's), and eosinophilic granulomatosis with polyangiitis (EGPA instead of Churg-Strauss Syndrome).

ANCA associated vasculitides are a group of small to medium vessel inflammatory diseases that cause end organ damage from vessel thrombosis that results from inflammation of the vascular endothelial lining. Ophthalmic manifestations are protean and nonspecific and are secondary to vasculitis of the ophthalmic circulation. GPA patients have a higher likelihood of ocular or orbital involvement than EGPA patients [65, 66].

Conjunctivitis, episcleritis, and scleritis are the most common presentations. The patients typically present with painful red eye except in episcleritis where the condition might be painless and usually self-limiting. Conjunctivitis can sometimes present with granulomas that are often bilateral. Cicatrizing conjunctivitis resulting in symblepharon more commonly of the upper eyelid can be seen in uncontrolled disease [67, 68]. Scleritis associated with systemic vasculitis requires more aggressive treatment than idiopathic scleritis or from other etiologies [69].

Cornea can be affected in the form of exposure keratitis as a result of conjunctival scarring or more seriously as peripheral ulcerative keratitis (PUK). GPA is the second most common cause of PUK after rheumatoid arthritis [70] and has a similar presentation.

Vasoocclusive phenomenon of the retinal artery or veins is uncommon but has been described as case reports in both GPA and EGPA [71–75]. Patients present with sudden, painless loss of vision. Retinal arterial involvement can be in the form of central retinal artery occlusion or a branch of it. Involvement of posterior ciliary circulation can cause ischemic optic neuritis.

Rarely patients with ANCA vasculitis can present with anterior, posterior, or panuveitis. Uveitis tends to occur in less than 10% of the cases [67, 76, 77] and is thought to be a result of spill-over of inflammation from adjacent scleritis (which can sometimes be the heralding manifestation) or keratitis [78]. No gender differences have been reported in the incidence or disease course of ocular manifestations seen with systemic vasculitides.

## 7. Behçet's Disease

Behçet's disease (BD) is a chronic multiorgan occlusive vasculitis that affects venules more than arteries but can affect vessels of any size [79, 80]. It is most prevalent in countries along the historic Silk Route with Far and Middle East having a higher prevalence than Europe [81, 82]. In some countries such as Turkey the prevalence is as high as 80–300 cases per 100,000 [82]. Hence, much of the current literature comes from studies done in Turkey, Iran, and Japan. It usually presents with recurrent oral and genital ulcers, cutaneous inflammation, and relapsing uveitis usually in the third decade of life with male predominance noted in most studies [82–85]. International Study Group for Behçet's Disease (ICBD) has included ocular involvement as part of the diagnostic criteria [86].

Uveitis is the most common ocular manifestation affecting more than 50–80% of patients with BD [87]. Bilateral panuveitis is the most frequent form irrespective of the patient age group [82, 83]. Behçet disease associated uveitis carries a poor visual prognosis with more than a quarter ending up legally blind [83, 88]. Male gender has been proposed as a risk factor for developing BD related uveitis [89, 90] and has also been found to be associated with worse outcomes in terms of disease course and response to therapy [91, 92]. This is thought to be secondary to a higher incidence of posterior segment involvement and retinal vasculitis in males compared to females. Other prognostic factors include duration of uveitis and age at onset.

Like all autoimmune conditions, genetics plays an important role in the disease pathogenesis. Human leukocyte antigens (HLA-) B51 and HLA-A26 have been identified as predisposing factors associated with BD related uveitis. HLA-A26 has also been proposed to be associated with worse visual outcomes by increasing the risk of posterior involvement [93, 94] whereas HLA-B51 is associated with earlier onset age of uveitis and male gender [95] in patients with posterior uveitis.

Hence male gender serves not only as a risk factor for developing uveitis in patients with Behçet's disease but also as a marker of poor visual outcome once the uveitis develops.

## 8. Sarcoidosis

Sarcoidosis is characterized by multisystem noncaseating granulomas that result from an exaggerated immune response to self or non-self-antigens [96]. No specific etiology has been identified. Genetics (HLA-B8 is the most common allele that has been associated with sarcoidosis) [97] and infectious agents (mycobacteria, human herpes virus 8) [98, 99] as well as environmental factors have been implicated in the pathogenesis. Inflammation can involve multiple organ systems with the most common ones being lymph nodes, lungs, eyes, and skin. Ocular sarcoidosis can be seen in up to 25–50% of the patients with systemic disease. This can be the unmasking feature leading to the diagnosis [100]. The incidence also varies with race. African-Americans are 10–20 times more commonly affected than Caucasians [101]. It affects both genders similarly with a slight female predominance [102]. A bimodal incidence has been reported with the first peak between the 2nd and 3rd decade and the second between the 5th and 6th decade of life [103, 104].

Bilateral granulomatous uveitis can sometimes be the only sarcoid related pathology. Bilateral ocular involvement is the most common ocular pattern occurring in 30–70% of the cases [105–108]. Anterior uveitis is the most common type; however distribution of uveitis may vary with race. Some studies have reported posterior or panuveitis to be more common in Caucasians compared to African-Americans [109–112]. The International Workshop on Ocular Sarcoidosis (IWOS) has identified seven signs “suggestive” for the diagnosis of ocular sarcoidosis. These include (1) mutton-fat/granulomatous keratic precipitates and/or iris nodules (Koeppe/Busacca), (2) trabecular meshwork nodules and/or tent-shaped peripheral anterior synechiae, (3) snowballs/string of pearls vitreous opacities, (4) multiple chorioretinal peripheral lesions (active and/or atrophic), (5) nodular and/or segmental periphlebitis (+/− candlewax drippings) and/or retinal macroaneurysm in an inflamed eye, (6) optic disc nodule(s)/granuloma(s) and/or solitary choroidal nodule, and (7) bilaterality [113].

About 10% of sarcoid associated uveitis cases can have significant visual dysfunction and even blindness [114]. Female gender, Caucasian race, late onset of systemic disease, and presence of multifocal choroiditis or panuveitis have been associated with a worse visual outcome [109, 115–117]. White patients had more posterior segment involvement and white females had frequent occurrence of cystoid macular edema and therefore worse visual outcomes [117].

Keratoconjunctivitis sicca (KCS) is the second most common ocular manifestation followed by adnexal granulomas. KCS or dry eye syndrome results not only from aqueous layer deficiency as a result of lacrimal gland inflammation leading to fibrosis but also from mucin and lipid layer deficiencies. The latter is in fact the main underlying pathology [118]. This is more common in women as mentioned earlier in the Rheumatoid Arthritis section. Clinically, significant lacrimal gland involvement resulting in enlargement of the lacrimal gland is an uncommon finding [112].

Adnexal granulomas most commonly affect the conjunctiva followed by eyelid. Rarely lacrimal gland and orbital granulomas are also seen [112]. According to IOWS diagnostic criteria, they can also be present in the trabecular meshwork (Berlin nodules) and cause an elevated intraocular pressure secondary to angle closure [113, 119]. Like elsewhere histopathology shows noncaseating granulomas and sometimes can help form the diagnosis.

Female gender acts as a prognostic factor related to worse visual outcomes in patients with ocular sarcoidosis.
